# Effectiveness of the CoronaVac® vaccine in a region of the Colombian Amazon, was herd immunity achieved?

**DOI:** 10.1186/s40794-021-00159-x

**Published:** 2022-01-15

**Authors:** Héctor Serrano-Coll, Hollman Miller, Camilo Guzmán, Ricardo Rivero, Bertha Gastelbondo, Jorge Miranda, Ketty Galeano, Jhon Montaña-Restrepo, Salim Mattar

**Affiliations:** 1 Universidad de Córdoba, Instituto de Investigaciones Biológicas del Trópico, Montería, Colombia; 2 Instituto Colombiano de Medicina Tropical-Universidad CES, Medellín, Colombia; 3 Secretaria de Salud del Vaupés, Mitú, Colombia

**Keywords:** COVID-19 vaccines, Prevention, Post-exposure, Prophylaxis, Public health, Mass vaccination

## Abstract

**Introduction:**

Currently, more than 4.5 billion doses of SARS-CoV-2 vaccines have been applied worldwide. However, some developing countries are still a long way from achieving herd immunity through vaccination. In some territories, such as the Colombian Amazon, mass immunization strategies have been implemented with the CoronaVac® vaccine. Due to its proximity to Brazil, where one of the variants of interest of SARS-CoV-2 circulates.

**Objective:**

To determine the effectiveness of the CoronaVac® vaccine in a population of the Colombian Amazon.

**Methods:**

Between February 24, 2021, and August 10, 2021, a descriptive observational study was carried out in which a population of individuals over 18 years of age immunized with two doses of the CoronaVac® vaccine was evaluated. The study site was in the municipality of Mitú, Vaupés, in southeastern Colombia, a region located in the Amazon bordering Brazil. Results. 99% of the urban population of the Mitú municipality were vaccinated with CoronaVac®. To date, 5.7% of vaccinated individuals have become ill, and only 0.1% of these require hospitalization. One death was attributable to COVID-19 has been reported among vaccinated individuals, and the vaccine has shown 94.3% effectiveness against mild disease and 99.9% against severe infection.

**Conclusions:**

The herd immunity achieved through mass vaccination in this population has made it possible to reduce the rate of complicated cases and mortality from COVID-19 in this region of the Colombian Amazon.

**Highlights:**

- CoronaVac® has shown 94.3% effectiveness against mild disease and 99.9% against severe infection in this indigenous population.
- CoronaVac® reduces the mortality rate from 2.2% in 2020 to 0.22% in 2021.
- The herd immunity was achieved through mass vaccination in this region of the Colombian Amazon.

## Introduction

Currently, around 168 million cases and more than three million deaths from Coronavirus disease 2019 (COVID-19) have been reported, and more than 4.5 billion doses of vaccines against SARS-CoV-2 have been applied worldwide (August 11, 2021) [1]. However, only 26.6% of its population has been fully immunized in developing countries such as Colombia, so herd immunity is still far from being achieved (August 11, 2021) [2]. The proximity to countries such as Brazil, where the appearance of the P.1 variant has endangered the health system of this country [3], Colombian Amazon was prioritized with the vaccination’s program.

Due to storage and transportation facilities, the CoronaVac® vaccine (Sinovac, China) was chosen for mass immunization in tropical regions of Colombia, such as the Amazon. This vaccine platform consists of a chemically inactivated SARS-CoV-2 virus and has proven to be safe, effective, and immunogenic against this new virus, and around 100 million doses of this vaccine have been applied worldwide [4]. Furthermore, this strategy of vaccination using CoronaVac® was used successfully in a small population in Serrana, Brazil [5]. Therefore, this vaccination strategy could be relevant to mitigate the spread of SARS-CoV-2 in small and remote communities in Latin America.

On the other hand, as of august 10, 2021, Colombia has received 13,299,364 vaccines against COVID-19; 7,872,675 (40.1%) from Sinovac, 7,872,440 (40.24%) from Pfizer-Biotech, 2,085,073 (10.66%) from AstraZeneca, 1,171,453 (5,99%) from Janssen, and 608.142 (3.11) From Moderna, and it is essential to note that of the total number of vaccines applied in this country to date, 40% corresponds to CoronaVac® [6].

This work aimed was to determine the effectiveness of the CoronaVac® vaccine in a population of the Colombian Amazon.

## Methods

**Fig. 1:**
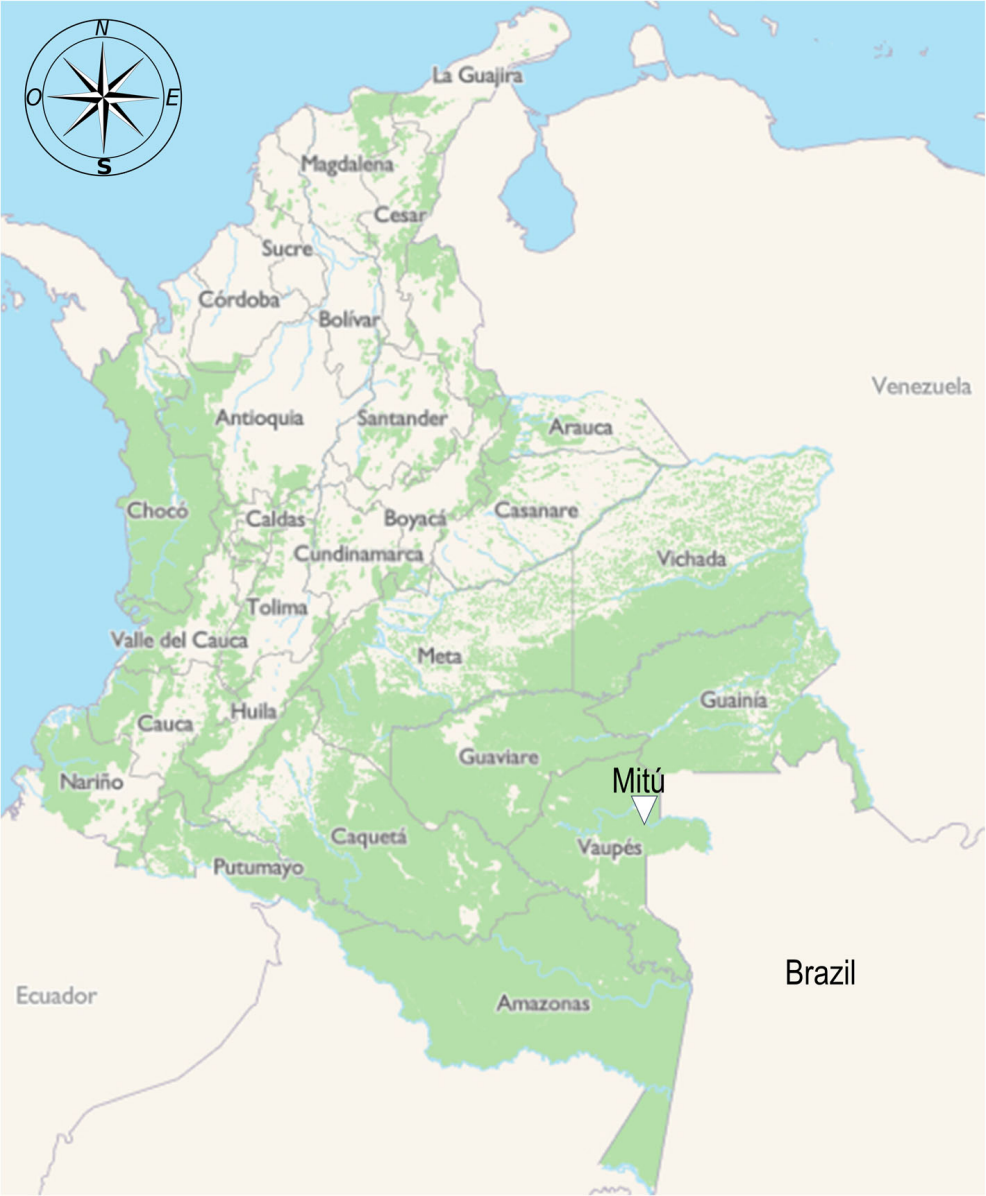
The geographic location of the municipality of Mitu. This figure showed that Mitu is located in the southeast of Colombia on the border with Brazil

A descriptive observational study was carried out in which a population of individuals older than 18 years immunized with two doses of the CoronaVac® vaccine (Sinovac, China) was evaluated. The study period was between February 24, 2021, to August 10, 2021. The work was developed in the municipality of Mitú, Vaupés, Colombia, a region located in the southeast of Colombia (Amazonas) bordering Brazil (Fig. 1). Mitú is the capital of Vaupés and has 7856 inhabitants, immunized with two doses with an interval of 20 days with the CoronaVac® vaccine that uses SARS-CoV-2 chemically inactivated with beta-Propiolactone [7, 8]. Sociodemographic and clinical characteristics and vaccination data of patients were obtained from secondary sources as a raw database supplied by the Mitu municipality’s health secretary. The primary outcome of this study was to evaluate the effectiveness of CoronaVac® in reducing mortality and severe illness due to SARS-CoV-2 in individuals with a complete vaccination schedule. On the other hand, the description of these outcomes was carried out through an active search for COVID-19 cases by the Mitu health secretary.

The disease’s severity was defined by the following criteria [9, 10]: A) Mild disease: local symptoms in the upper respiratory tract and may present with non-specific symptoms such as fever, pain muscle, or general discomfort. B) Moderate disease: clinical or radiological evidence of lower respiratory infection, with compatible lung images and O2 saturation > 93%, and C) Severe disease: respiratory rate greater than 30/min, oxygen saturation < 93%, PAFI (the relationship between arterial oxygen pressure and the inspired fraction of oxygen (PaO2 / FIO2) less than 300, infiltrates greater than 50%.

### Ethical aspects

The research was carried out following the international ethical standards given by the World Health Organization (WHO) and the Pan American Health Organization, supported by the Declaration of Helsinki and the Ministry of Health of Colombia resolution number 008430 of 1993 and endorsed by the Committee of Ethics of the Institute of Biological Research of the Tropic, University of Córdoba.

### Analysis of data

The data were analyzed by the biostatistics group of the Institute of Biological Research of the Tropic-University of Córdoba using the statistical package for the Social Sciences version 27 (SPSS) and the software GraphPad Prisma 8, and univariate analysis was performed. For qualitative variables, it was performed through the calculation of absolute and relative frequencies. The measures of central tendency were calculated as quantitative variables.

## Results

### Characteristics of the evaluated population

60.4% of the population of the municipality of Mitu is predominantly indigenous. Besides, 99.9% (7849 people) completed their vaccination schedule with two doses of CoronaVac®. Of those vaccinated, 45.3% were women and 54.7% men, the median age was 38 years and 84.6% were under 60 years of age, eight (0.1%) women were pregnant and voluntarily vaccinated (Table 1).

**Table 1:** Characteristic of the individuals vaccinated with two doses in Mitu municipality

Characteristic of the individuals vaccinated (%)	
**Sex**	
Female	3530 (45)
Male	4319 (55)
**Median age in years (range)**	38 (18–95)
Individuals < 60 years	6644 (84.6)
Individuals > 60 years	1205 (15.4)
**Ethnicity**	
Indigenous	4745 (60.4)
Afro-Colombian	156 (2)
Other	2948 (37.6)
**Pregnant women vaccinated**	
Yes	8 (0.1)
**Total of people with two doses**	7849 (99.9)

### Incidence of SARS-CoV-2 infections after vaccination

From March 23 to August 10, 2021, 447 cases have been presented, corresponding to 5.7% of vaccinated individuals (Table 2). Regarding the severity of the infection, the age range, under 60 years there were 406 infections, of these 405 (99.8%) were mild infections and one (0.2%) with moderate severity, and in those over 60 years, there were 41, of these 40 (97.6%) were mild infections and one (2.4%) was severe, and this individual died as a direct consequence of COVID-19 (Table 3).

**Table 2:** Characterization of the SARS-CoV-2 infected individuals post-vaccinated

Characteristic of the individuals infected (%)	
Female	230 (51.5)
**Male**	217 (48.5)
**Test used for SARS-CoV-2 diagnostic**	
Antigen	268 (60)
RT-qPCR	179 (40)
**Severity of COVID-19**	
Mild	445 (99.6)
Moderate	1 (0.2)
Severe	1 (0.2)
**Type of treatment**	
Ambulatory care	445 (99.6)
Hospitalized	2 (0.4)
**Deceased by COVID-19**	1 (0.2)
**Total of people infected with COVID-19**	447 (5.7)

**Table 3:** Severity of COVID-19 in population vaccinated according to age range <  60 years vs >  60 years

Severity of COVID-19 according to age range (%)	
< 60 years	*N* = 406
Mild	405 (99.8)
Moderate	1 (0.2)
Severe	0
**> 60 years**	*N* = 41
Mild	40 (97.6)
Moderate	0
Severe + deceased	1 (2.4)

**Fig. 2:**
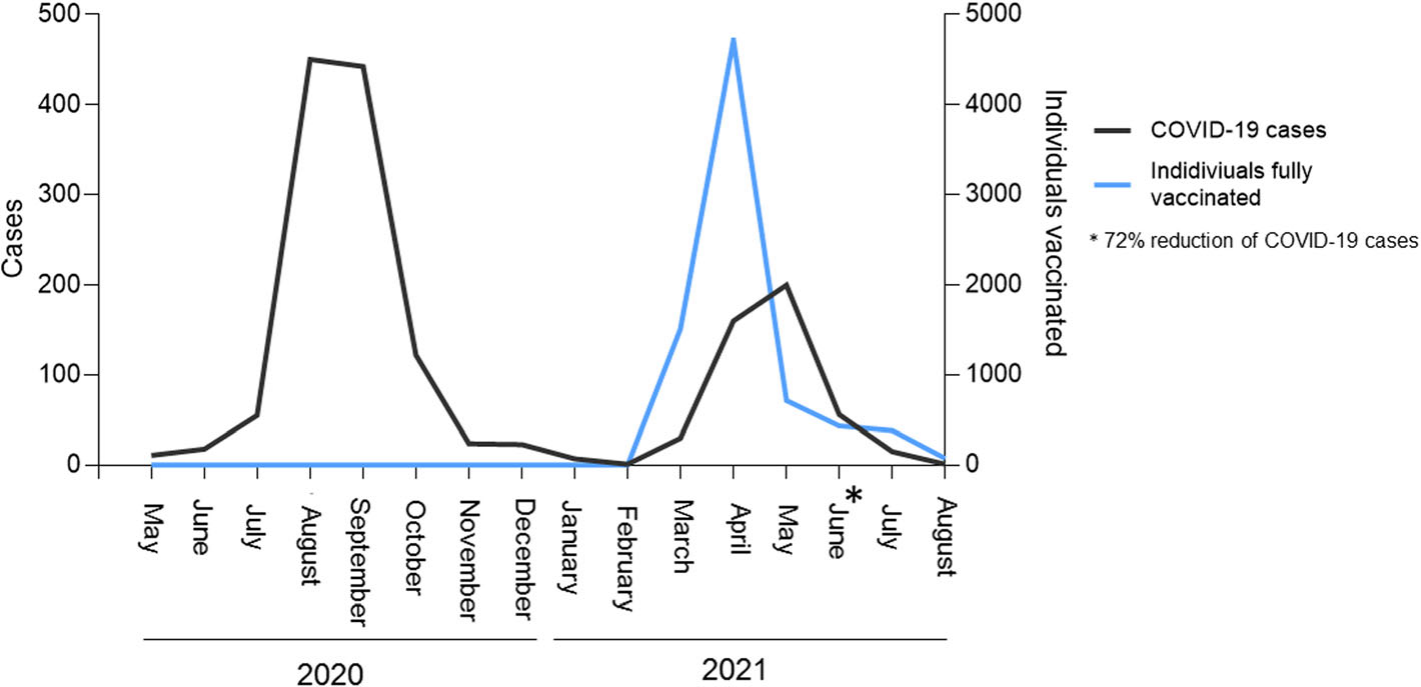
Characterization of COVID-19 cases in Mitu municipality. This figure showed a first peak or wave of cases of COVID-19 in August 2020, with a significant drop of cases in November 2020. The vaccination in this municipality started in February 2021 and a second wave was observed between April–May 2021, the highest peak of individuals who completed their CoronaVac® vaccination reduced COVID-19 cases by 72% in June

In May 2021, in Mitu, a new peak of SARS-CoV-2 was observed with 200 cases. This increase is much lower than the August 2020 peak, where 327 were reported. In addition, it can be observed that between April–May 2021, the highest peak of individuals who completed their CoronaVac® vaccination reduced COVID-19 cases by 72% in June (Fig. 2). On the other hand, when comparing the fatality rate, it was 2.2% before vaccination and 0.22% in the immunized population (Table 4).

**Table 4:** Effectiveness of the Coronavac vaccine

Effectiveness of CoronaVac	
Prevent mild forms	94.3%
Prevent moderate forms	99.9%
Prevent severe forms	99.9%
Prevent deaths	99.9%
Mortality rate pre-vaccination*	2.2%
Mortality rate post-vaccination in individuals fully vaccinated	0.22%

*Data obtained from DANE Colombia. (https://www.dane.gov.co/files/investigaciones/poblacion/defunciones-covid19/boletin-defunciones-covid-2020-02mar-2021-17ene.pdf)

### Vaccination effectiveness in the different forms of the severity of COVID-19

Regarding the vaccine’s effectiveness, it was observed that it was 94.3% to prevent mild forms and 99.9% for the case of moderate and severe forms. Besides, the vaccine was 99.9% effective in preventing cases of death attributed to SARS-CoV-2 has been reported among the vaccinated group (Table 4).

## Discussion

The vaccine demonstrated a significant of 94.3% efficacy in clinical trials for preventing SARS-CoV-2 infections in different stages of severity. With this efficacy, herd immunity may have been achieved through mass vaccination in this population. This vaccine’s effectiveness study in a predominantly indigenous population is similar in size to the phase III studies conducted in Turkey and Brazil, in which between 7000 and 13,000 participants were evaluated [11].

SARS-CoV-2 infections among those vaccinated were mild, and their management was ambulatory. In addition, it has been seen that vaccination with the immunogen from the pharmaceutical company Sinovac has prevented the appearance of complicated infections and fatal outcomes [12]. These findings are consistent with those reported by phase III studies carried out in Brazil, where it was shown that this vaccine reduces the risk of hospitalization and death between 84 to 100% of individuals vaccinated with CoronaVac® [12]. However, our results in the older than 60 years show differences with what was published in Brazilian older adults by Ranzani et al. [13], who found protection of 49.4%. The vaccine’s reduction could be explained because 83% of their cases were infected with the P.1 variant of SARS- CoV-2.

Furthermore, it is essential to analyze the course of infection over time and the impact of vaccination against SARS-CoV-2. In April 2021, the third wave of COVID-19 cases began in Colombia. However, the incidence was much lower than observed in the first peak of the pandemic between April and June 2020. The new cases presented in 2021 in the vaccinated population could be due to the Brazilian variant P.1 of SARS-CoV-2 [14]. However, the morbidity and mortality of this new variant seem to be controlled with the CoronaVac® vaccine.

Regarding the effectiveness of this vaccine, it was observed that it was 94.3% against mild disease and 99.9% against severe infection in this population. Our findings are similar to Turkey’s phase III study for CoronaVac®, in which efficacy of 91% was observed. In contrast to studies in Brazil and Chile, which reported low overall efficacy of 50.38 and 65%, respectively. However, it is essential to highlight that this vaccine reduced 90% of the proportion of hospitalization in an intensive care unit (ICU) and 86%mortality from SARS-CoV-2 [15, 16] in the Chilean population. The epidemiological moments of vaccination must also be taken into account. For example, Chile began vaccination with a low viral transmission different from the epidemiological scenario studied in Brazil. When the transmission is lower, there is less chance that vaccination will fail [17]. Our study is similar to perform in the small city of Serrana, Brazil, that vaccinated using CoronaVac®. In Serrana, 95% of the city’s adult population was vaccinated, a reduction of 80% in symptomatic cases and hospitalizations dropped by 86% and mortality by 95% [5].

So far, SARS-CoV-2 is a virus that is efficiently transmitted and quickly infects the unvaccinated population. Due to the lack of genotypic information for the Mitú municipality, we do not know if the P1 variant (Brazil) managed to spread or if the action of the vaccine contained it. On the other hand, one of the limitations of this work could be in a possible under-registration of the mild infections registered in this vaccine population, since it was not possible due to the type of study that was proposed to carry out a strict follow-up by RT- qPCR to this population cluster.

The primary outcome of this study was to evaluate the effectiveness of CoronaVac® in reducing mortality and severe illness due to SARS-CoV-2. On the other hand, one of the limitations of this work could be in a possible under-registration of the mild infections registered in this vaccine population, since it was not possible due to the type of study that was proposed to carry out a strict follow-up by RT- qPCR to this population cluster.

Finally, we can infer that to date, herd immunity has been achieved through mass vaccination in this population, which has impacted the reduction of complicated cases and the mortality rate from COVID-19. However, pediatric populations remain unvaccinated, which could cause few breakthrough infections with an increase in the number of cases at a given epidemiological moment. It is also necessary to know if the CoronaVac® will protect against the new delta strain in Colombia. It will be a real challenge for the vaccine in a couple of months when it is believed that Delta could be predominant in Colombia. Public health must continue long-term surveillance to measure the effect of vaccination in the studied population. It is unknown if the vaccine’s immunity will be maintained over time and if a booster of this immunogen is needed in the short or medium term. There is still a long way to walk on this exciting research topic that will be key to controlling and mitigating the pandemic caused by SARS-CoV-2.

## Data Availability

The raw data supporting the conclusions of this article will be made available by the authors, without undue reservation, to any qualified researcher.

## Abbreviations

COVID-19: Coronavirus Disease 2019
WHO: World Health Organization
ICU: Intensive Care Unit
